# Non-Breeding Song Rate Reflects Nutritional Condition Rather than Body Condition

**DOI:** 10.1371/journal.pone.0036547

**Published:** 2012-05-09

**Authors:** Alain J.-M. Van Hout, Rianne Pinxten, Ann Geens, Marcel Eens

**Affiliations:** Research Group of Ethology, University of Antwerp, Wilrijk, Belgium; Utrecht University, The Netherlands

## Abstract

Numerous studies have focused on song in songbirds as a signal involved in mate choice and intrasexual competition. It is expected that song traits such as song rate reflect individual quality by being dependent on energetic state or condition. While seasonal variation in bird song (i.e., breeding versus non-breeding song) and its neural substrate have received a fair amount of attention, the function and information content of song outside the breeding season is generally much less understood. Furthermore, typically only measures of condition involving body mass are examined with respect to song rate. Studies investigating a potential relationship between song rate and other indicators of condition, such as physiological measures of nutritional condition, are scant. In this study, we examined whether non-breeding song rate in male European starlings (*Sturnus vulgaris*) reflects plasma metabolite levels (high-density lipoproteins (HDL), albumin, triglycerides and cholesterol) and/or body mass. Song rate was significantly positively related to a principal component representing primarily HDL, albumin and cholesterol (and to a lesser degree plasma triglyceride levels). There was only a trend toward a significant positive correlation between song rate and body mass, and no significant correlation between body mass and the abovementioned principal component. Therefore, our results indicate that nutritional condition and body mass represent different aspects of condition, and that song rate reflects nutritional rather than body condition. Additionally, we also found that intra-individual song rate consistency (though not song rate itself) was significantly positively related to lutein levels, but not to body mass or nutritional condition. Together our results suggest that the relation between physiological measures of nutritional condition and song rate, as well as other signals, may present an interesting line of future research, both inside and outside the breeding season.

## Introduction

Numerous studies have focused on the information content of animal signals, particularly in birds species [1], [2]. Notably, bird song has received much attention from researchers as a signal involved in mate choice and intrasexual competition [3], [4], [5], [6], [7]. The information conveyed by song has therefore, for the most part, been examined during the breeding season, when song expression is often enhanced due to seasonally elevated plasma testosterone levels [6] and thought to express individual condition or quality [2]. By contrast, while a fair number of studies have examined seasonal variation in bird song and its neural substrate [8], [9], and for some species, such as the European starlings (*Sturnus vulgaris*), much progress has been made in understanding the underlying neural and hormonal mechanisms [10], [11], [12], the function and information content of non-breeding song has received little attention and is generally much less understood [13]. It has, however, been suggested that in at least some species non-breeding song may also affect mating decisions and pair formation, as well as social cohesion and territorial dominance [5], [14], [15]. Therefore, understanding the information content of non-breeding song may prove essential to form an integrated picture of seasonal variation in the expression and physiological control of song as a signal.

The amount of singing activity, typically referred to as song rate, is expected to be dependent on neuromuscular capacities and performance abilities [6], as well as an individual’s energetic state and condition, which can reflect their energy reserves, their ability to sequester resources and/or the efficiency of underlying physiological pathways [6], [16], [17]. Additionally, Hill [17] recently proposed that “a condition-dependent display trait is a conspicuous feature of an organism that varies in expression depending on the capacity to withstand environmental challenges”. For dynamic traits such as song rate, it could therefore be expected that not only the degree of expression of the trait may be condition-dependent, but also the amount of variation it may display over time and/or under varying conditions.

From a theoretical perspective it would be expected that the possession of greater energy reserves (as an aspect of better condition) would allow for a greater energy investment towards ‘non-maintenance behaviors’, such as singing (see [16] for discussion). Observations of a positive correlation between song rate and body mass, as well as the positive effects of food supplementation on song rate support this expectation ([7], [16], but see [18]). However, typically only body mass (or its residual against body size) is used as a measure of condition [7], [16]. Studies investigating a potential relationship between song rate and other aspects of condition, such as physiological measures of nutritional condition, are scant [6]. These plasma metabolites can however provide an insight into conditions over time through a single time point measure [19]. At the same time, being closely linked to dietary state [20], some plasma metabolites may also represent a dynamic measure of condition [21]. For instance, plasma albumin and triglycerides respectively represent protein and lipid reserves [19], [20], [22], while plasma levels of cholesterol, an important precursor to e.g. steroid hormones such as testosterone, may also reflect energetic state [23]. Plasma lipoproteins such as high-density lipoproteins (HDL) furthermore enable triglycerides and cholesterol to be transported within the water-based bloodstream [24], [25]. Most of these plasma metabolites have been used extensively as nutritional condition parameters for birds [19], [22], [26].

European starlings are seasonally breeding songbirds, the males of which produce high levels of song even when plasma T levels are basal, such as during fall (the non-breeding season) [10], [27] as well as after castration [28], and the females of which can also produce song through out the year [29]. These findings make the European starling one of only a few temperate climate songbirds species who, apart from during the breeding season, also display a high song rate throughout most of the non-breeding season [5]. Studies involving European starlings have, among other things, provided important insights into the physiological and neural basis of song outside the breeding season, and how this relates to breeding season song [8], [10], [11], [12]. However, surprisingly little is know about the function and information content of this non-breeding song. Here, we used captive male European starlings to investigate whether non-breeding song (during fall) conveyed information about individual condition. Therefore, we determined (a) whether song rate reflected nutritional condition, measured through plasma metabolite levels, and (b) whether this potential relation is dependent on a correlation between body mass and song rate, and between body mass and nutritional condition, respectively. We furthermore also investigated whether intra-individual variation over time with respect to song rate may be explained by differences in condition. Finally, since we previously observed a positive effect of experimental carotenoid supplementation on song rate [30], the relation between plasma carotenoid (i.e. lutein) levels and song rate, as well as intra-individual variation in song rate, was also examined.

## Materials and Methods

### Ethics Statement

The housing and experimental procedures were performed in agreement with the Belgian and Flemish laws and were approved by the ethical committee of the University of Antwerp (ID number 2006/22). European starlings have been shown to adapt easily to captivity and to show normal social behavior under these conditions [5], [27]. Both aviaries used in this study were equipped with 20 identical nest boxes, each equipped with a 30 cm perch allowing the males to sit or sing in front of it, several larger perches, shelter, and food and water ad libitum. When taking blood samples, care was taken to minimize stress for the starlings.

### Origin and Housing of Starlings

The 37 male starlings used in this study were captured as juveniles. At the time of the study, they were between 2 and 4 years old and housed (pseudo-randomly with respect to age) in two large aviaries (N = 18 and N = 19; University of Antwerp, Belgium), equipped with ample nest boxes, perches, shelter, and food and water ad libitum [30]. The bill color of all starlings remained black throughout the course of the study, which represents a highly sensitive and integrated measure of T concentration and action over a period of several days [27], [31], [32], indicating that plasma T levels of all starlings had remained basal.

### Body Mass and Metabolites

On 25 November 2008, body mass and tarsus length was determined (to the nearest 1 g and 0.1 mm, respectively) and blood samples were collected (approximately 500 µL, extracting plasma after 10 min centrifugation at 1500 g). As the residuals of body mass and tarsus length were highly correlated with body mass itself (r_37_ = 0.84, p<0.0001), body mass was used as a more direct measure of condition. Plasma metabolites levels (albumin, triglycerides, cholesterol and HDL) were determined using a Horiba ABX Mira Chemistry Analyser (Horiba, France; see [26]). Repeatability (within-run precision) and reproducibility (run-to-run precision) of the measurements are certified by including a calibrator and a control (provided by Horiba ABX) with known concentrations each run and was in all cases between 95 and 105% of the certified value. Plasma lutein levels were measured as described in detail by [30]. As the amount of plasma varied, not all parameters could be determined for all individuals.

### Observations

Prior to the blood sampling, we used a point sampling technique with a one minute interval to monitor the behavior of all starlings simultaneously (as described elsewhere: [27]), in three sessions of approximately 45 min, all between 09h00 and 13h00 on the 22nd, 23rd and 24th November 2008, while alternating the order of the aviaries between subsequent days. Song rate was defined as the proportion of samples during which a male was singing compared to the total number of samples. This measure is repeatable (this study: r = 0.22, p<0.0001; [33]), even between seasons [27]. The mean of these 3 samples was used as the individual measure of song rate in further analyses. For each individual we also determined the standard deviation of the song rate measurements across the different observation days (as a measure of song rate variability). As this measure was positively correlated with song rate (Pearson correlation test: r_37_ = 0.352, p = 0.03), this shared variation was statistically removed by determining the residuals of the starlings’ song rate standard deviations (when controlling for song rates). These were subsequently multiplied by −1 (in order to invert the orientation of the axis) to obtain a measure for individual ‘song rate consistency’.

### Statistical Analyses

Statistical analyses were performed in XLSTAT 2010™ (Addinsoft, USA). Normal distribution was confirmed using Shapiro-Wilk tests. As song rate represents a fraction, arcsine-square root-transformed song rate data were used in the following analyses. Initial Pearson correlation tests showed that HDL, albumin and cholesterol, and to a lesser degree triglycerides, are all strongly inter-correlated (Table 1). Because of this observation, and also to avoid multiple testing, a principal component analysis (PCA) was performed for these nutritional condition parameters (however, individual correlations are also reported and addressed in the discussion). A single principal component (PC) with an eigenvalue larger than one was obtained (eigenvalue: 2.348), explaining 59% of the total variation (factor loadings reported in Table 1). This PC was used in a linear regression model with song rate as the dependent factor. Potential aviary and age effects were non-significant (p>0.13) and therefore not included in the final model. A linear regression with the PC as an independent factor was used to assess whether body mass also reflects plasma metabolite levels (i.e. the PC), while a Pearson correlation test was used to examine whether song rate reflects body mass. The same approach was applied to investigate whether song rate consistency was dependent on either plasma metabolite levels (i.e. the PC) or body mass. For plasma lutein levels, the same approach was used as for body mass. For all tests, an α of 0.05 was used to judge significance.

**Table 1:** Intercorrelations of plasma levels of HDL, albumin, cholesterol and triglycerides and their factor loadings for the principal components of nutritional condition.

	HDL	Albumin	Cholesterol	Triglycerides	Factor loadings
HDL	–	0.59***	0.72***	0.53**	0.954
Albumin		–	0.43**	0.18	0.740
Cholesterol			–	0.09	0.778
Triglycerides				–	0.534

** p<0.01;

*** p<0.001.

## Results

### Song Rate, Plasma Metabolites and Body Mass

**Figure 1:**
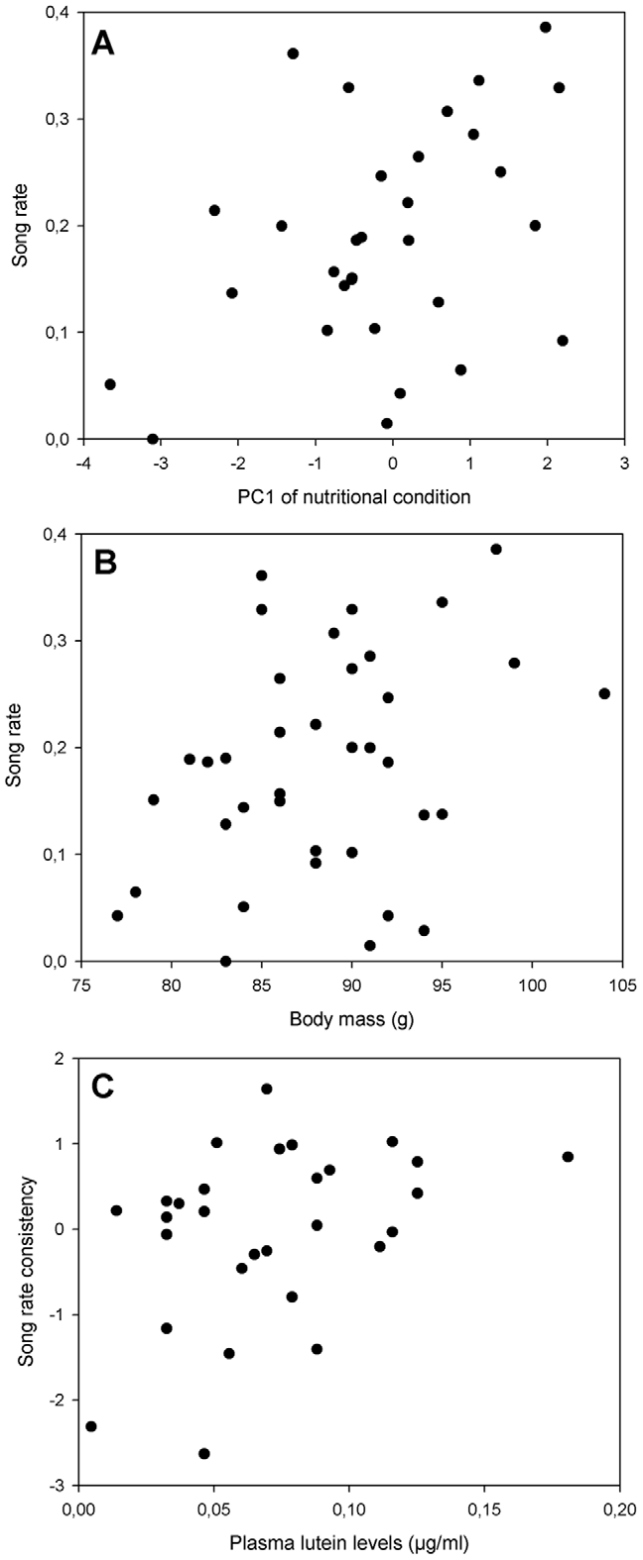
Correlations between song rate and nutritional condition, body mass and plasma lutein levels. (A) Between average song rate and the PC of nutritional condition, reflecting primarily HDL, albumin and cholesterol (and to a lesser degree plasma triglyceride levels), (B) Between average song rate and body mass, and (C) Between song rate consistency and plasma lutein levels. Statistics are discussed in the text.

A linear regression with song rate as the dependent variable showed a significant positive effect of the PC (primarily HDL, albumin and cholesterol; β = 0.431, p = 0.02; Figure 1A; see also Table 2 for individual correlations). There was only a trend toward a significant positive correlation between song rate and body mass (r_37_ = 0.279, p = 0.095; Figure 1B). A linear regression model for body mass showed no significant effect of the PC (β = 0.262, p = 0.12). For song rate consistency, no significant effect of the PC was found (β = 0.131, p = 0.5), nor was song rate consistency significantly correlated with body mass (r_37_ = 0.21, p = 0.2).

**Table 2:** Pair-wise Pearson correlations of song rate and song rate consistency with plasma levels of HDL, albumin, cholesterol, triglycerides and lutein, and with body mass, respectively.

		HDL	Albumin	Cholesterol	Triglycerides	Lutein	Bodymass
Song rate	r	0.409	0.384	0.394	0.173	−0.040	0.279
	p	0.02	0.04	0.02	0.3	0.8	0.095
Consistency	r	0.210	−0.181	0.230	0.298	0.380	0.205
	p	0.25	0.28	0.18	0.08	0.04	0.22

### Song Rate and Plasma Lutein Levels

Plasma lutein levels were significantly positively correlated with song rate consistency (r_29_ = 0.38, p = 0.04; Figure 1C), but not with song rate (r_29_ = −0.04, p = 0.8). Using plasma lutein levels as a dependent variable, a linear regression model showed no significant effect of the PC (β = 0.083, p = 0.7).

## Discussion

**Figure 2:**
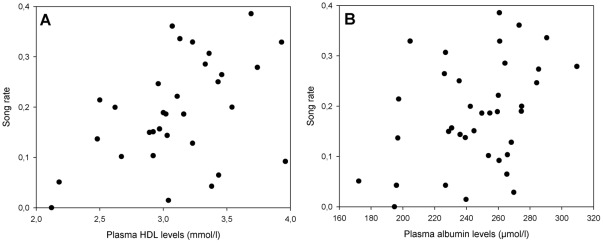
Correlations between song rate and separate plasma metabolite levels. (A) Plasma HDL levels (r_32_ = 0.45, p = 0.01), and (B) Plasma albumin levels (r_37_ = 0.37, p = 0.02).

Besides producing high levels of song rate during the breeding season, male European starlings typically also display a high song rate outside the breeding season [10], [27] as well as after castration [28]. Particularly in this species, a sizeable amount of studies have provided import insights into the physiological and neural basis underlying variation in song rate, both inside and outside the breeding season [8], [10], [11], [12]. The function and information content of song rate outside the breeding season is, however, not well understood. Notably, our results show that non-breeding song rate can reflect physiological parameters of nutritional condition (i.e. plasma metabolites), and in particular plasma levels of HDL, albumin and cholesterol (Table 1; see also Table 2 and Figure 2 for individual correlations). Given that all the factors of the PC (HDL, albumin and cholesterol, and to a lesser degree triglycerides) are strongly inter-correlated (Table 1), this suggests that to a large extent they reflect a singular aspect of condition.

At the time the results presented in the current study were obtained (during fall, the non-breeding season), plasma testosterone levels were basal [27], which was also evidenced by the lack of any change in bill coloration from black to yellow (as discussed in the Methods section; [27], [31], [32]). Therefore, it is unlikely that the observed relation between song rate and the measured nutritional condition parameters is in any way dependent on the previously mentioned positive effect of an experimental increase in plasma testosterone levels on song rate [30]. It would however be interesting for future studies to explore whether (or to what degree) both effects might interact.

Given the positive correlation between song rate and the PC (of which HDL shows the highest factor loading: Table 1, as well as the largest correlation: Table 2 and Figure 2A), and the fact that plasma HDL levels in humans show high heritability [34], HDL may be particularly interesting for future studies as a potential measure of condition and/or individual quality [21]. Although further research is required, HDL’s positive relation to song rate might involve its role in the mobilization of e.g. cholesterol and triglycerides within the bloodstream [25]. This would be in line with the notion that condition-dependence of ornamental traits may not only reflect an individual’s energy reserves, but could also reflect the efficiency of physiological pathways (as recently emphasized by Hill [17] and referred to as the Pathway Functionality Hypothesis). This connection of plasma HDL levels to condition is furthermore supported by the negative effects that immune challenges can have on plasma HDL levels [24], [35].

Along the same lines, HDL is also implicated in carotenoid mobilization (uptake, transport and delivery) [36]. However, although we previously experimentally demonstrated a positive effect of carotenoid supplementation on song rate [30]), here we did not find a significant relation between the PC of nutritional condition (i.e. HDL) and plasma lutein levels, nor between lutein levels and song rate. This suggests that the effect of carotenoids on song rate may itself depend on inter-individual variation in other physiological factors involved in carotenoid metabolism, or confounded by the effects of other aspects of condition (such as immune function) on HDL and plasma carotenoids levels [35].

The positive relation between song rate and the PC may also depend on the contribution to the PC of albumin and (to a lesser degree) triglyceride levels, which represent energy reserves under the form of proteins and lipid reserves, respectively [19], [20], [22]. Therefore, higher albumin or triglyceride levels may allow allocation of more energy towards non-maintenance, fitness-enhancing traits [21], which may include prolonged singing activity [16]. Similarly, the positive contribution of cholesterol to the PC also indicates that song rate may reflect energetic state [23]. Notably, this result was not dependent on individual differences in food availability (e.g. foraging ability or territory quality [16]), as the captive starlings received ad libitum food. Therefore, this finding suggests that individual differences in song rate may reflect inherent physiological differences, in accordance with the Pathway Functionality Hypothesis [17].

Our results show no significant correlation between body mass and the PC (primarily HDL, albumin and cholesterol). Therefore, this suggest that HDL, albumin and cholesterol levels (and to a lesser degree triglyceride levels) on the one hand and body mass on the other may represent two separate aspects of condition. Furthermore, our results show no significant correlation between song and body mass, the latter being an often-used index of condition [7], [19]. Our results could arguably also be interpreted as a trend for a positive correlation between song rate and body mass. However, in either case our data clearly indicates that song rate better reflects plasma parameters of nutritional condition than it reflects body condition.

Finally, contrary to song rate itself, song rate consistency did not significantly reflect either nutritional condition (i.e. the PC) or body mass. Song rate consistency however did significantly positively correlate with plasma lutein levels. Similarly to the abovementioned finding that experimental carotenoid supplementation positively affects song rate in European starlings [30], this also suggest a relation between singing activity and carotenoid metabolism. However, further research on this subject is needed.

In conclusion, our main results show that song rate in European starlings reflects information about nutritional condition rather than body condition, while the lack of a significant correlation between both indices of condition suggests that they represent distinct aspects of condition. It would be important for future studies to investigate whether this relation between song rate and nutritional condition also exists during the breeding season and in other species. Examining relationships between other (and/or multiple) signals and these plasma metabolites also presents an interesting line of future research [22].
